# Methyl 2-(5-fluoro-3-methyl­sulfinyl-1-benzofuran-2-yl)acetate

**DOI:** 10.1107/S1600536809030451

**Published:** 2009-08-08

**Authors:** Hong Dae Choi, Pil Ja Seo, Byeng Wha Son, Uk Lee

**Affiliations:** a Department of Chemistry, Dongeui University, San 24 Kaya-dong Busanjin-gu, Busan 614-714, Republic of Korea; b Department of Chemistry, Pukyong National University, 599-1 Daeyeon 3-dong, Nam-gu, Busan 608-737, Republic of Korea

## Abstract

In the title compound, C_12_H_11_FO_4_S, the O atom and the methyl group of the methyl­sulfinyl substituent lie on opposite sides of the plane of the benzofuran fragment [O—S—C—C and C—S—C—C torsion angles = 126.70 (13) and −123.55 (13)°, respectively]. The crystal structure is stabilized by weak non-classical inter­molecular C—H⋯O hydrogen-bond inter­actions. The crystal structure also exhibits aromatic π–π stacking inter­actions between furan/benzene and benzene/benzene rings of adjacent benzofuran ring systems [centroid–centroid distances = 3.8258 (9) and 3.8794 (9) Å] and a weak inter­molecular C—H⋯π ring inter­action.

## Related literature

For crystal structures of similar methyl 2-(5-halo-3-methyl­sulfinyl-1-benzofuran-2-yl)acetate derivatives. see: Choi *et al.* (2008*a* ▶,*b* ▶). For the pharmacological properties of benzofuran compounds, see: Howlett *et al.* (1999 ▶); Twyman & Allsop (1999 ▶).
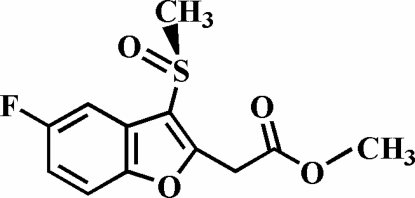


## Experimental

### 

#### Crystal data

C_12_H_11_FO_4_S
*M* *_r_* = 270.27
Triclinic, 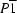

*a* = 7.7799 (5) Å
*b* = 8.5609 (6) Å
*c* = 10.5592 (7) Å
α = 73.834 (1)°
β = 80.178 (1)°
γ = 67.486 (1)°
*V* = 622.36 (7) Å^3^
*Z* = 2
Mo *K*α radiation
μ = 0.28 mm^−1^
*T* = 273 K
0.60 × 0.40 × 0.40 mm

#### Data collection

Bruker SMART CCD diffractometer
Absorption correction: none
5368 measured reflections
2667 independent reflections
2389 reflections with *I* > 2σ(*I*)
*R*_int_ = 0.014

#### Refinement

*R*[*F* ^2^ > 2σ(*F* ^2^)] = 0.032
*wR*(*F* ^2^) = 0.089
*S* = 1.08
2667 reflections
165 parameters
H-atom parameters constrained
Δρ_max_ = 0.28 e Å^−3^
Δρ_min_ = −0.31 e Å^−3^

### 

Data collection: *SMART* (Bruker, 2001 ▶); cell refinement: *SAINT* (Bruker, 2001 ▶); data reduction: *SAINT*; program(s) used to solve structure: *SHELXS97* (Sheldrick, 2008 ▶); program(s) used to refine structure: *SHELXL97* (Sheldrick, 2008 ▶); molecular graphics: *ORTEP-3* (Farrugia, 1997 ▶) and *DIAMOND* (Brandenburg, 1998 ▶); software used to prepare material for publication: *SHELXL97*.

## Supplementary Material

Crystal structure: contains datablocks global, I. DOI: 10.1107/S1600536809030451/jj2003sup1.cif

Structure factors: contains datablocks I. DOI: 10.1107/S1600536809030451/jj2003Isup2.hkl

Additional supplementary materials:  crystallographic information; 3D view; checkCIF report

## Figures and Tables

**Table 1:** Hydrogen-bond geometry (Å, °)

*D*—H⋯*A*	*D*—H	H⋯*A*	*D*⋯*A*	*D*—H⋯*A*
C3—H3⋯O4^i^	0.93	2.39	3.303 (2)	166
C9—H9*A*⋯O4^ii^	0.97	2.22	3.179 (2)	168
C9—H9*B*⋯O1^iii^	0.97	2.54	3.489 (2)	166
C12—H12*A*⋯O3^iv^	0.96	2.60	3.478 (2)	152
C11—H11*A*⋯*Cg*2^v^	0.96	2.97	3.93	173

Symmetry codes: (i) 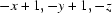; (ii) 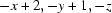; (iii) 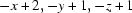; (iv) 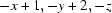; (v) 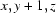. *Cg*2 is the centroid of the C2–C7 ring.

**Fig. 1.**
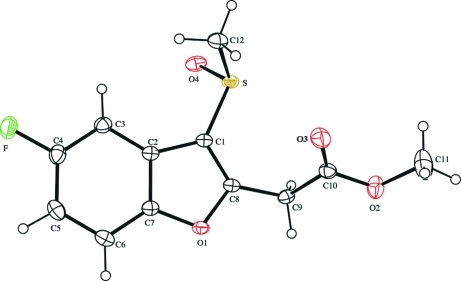
The molecular structure of the title compound with the atom numbering scheme. Displacement ellipsoids are drawn at the 30% probability level. H atoms are presented as a small cycles of arbitrary radius.

**Fig. 2.**
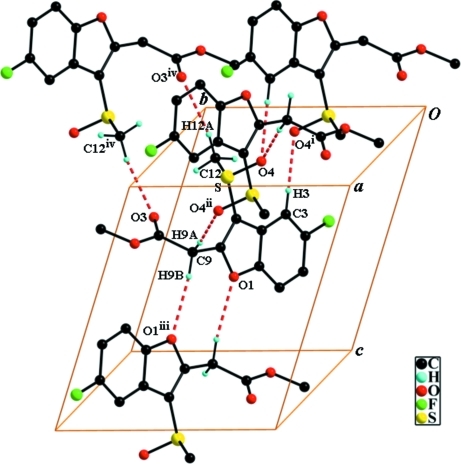
Weak, C–H···O hydrogen bond interactions (dotted lines) in the title compound. [Symmetry code: (i) - x + 1, - y + 1, - z; (ii) - x + 2, - y + 1, - z; (iii) - x + 2, - y + 1, - z + 1; (iv) - x+1, - y + 2, - z.]

**Fig. 3.**
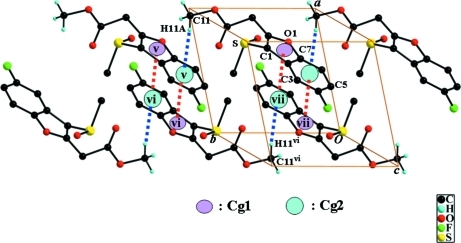
Weak, π–π stacking and C–H···π ring intermoleclar interactions (dotted lines) in the title compound. Cg1 and C2 denote ring centroids for C1/C2/C7/O1/C8 and C2-C7, respectively.[Symmetry code: (v) x, y + 1, z; (vi) - x + 1, - y + 2 , - z + 1; 1-x, 2-y, (vii) - x + 1, - y + 1, - z + 1.]

## supplementary crystallographic information

### Comment

Molecules containing the benzofuran skeleton have received considerable attention related to their pharmacological properties (Howlett *et al.*, 1999; Twyman & Allsop, 1999). This work is related to our communications on the synthesis and crystal structures of methyl 2-(5-halo-3-methylsulfinyl-1-benzofuran-2-yl)acetate derivatives, viz. methyl 2-(5-chloro-3-methylsulfinyl-1-benzofuran-2-yl)acetate (Choi *et al.*, 2008*a*), and methyl 2-(5-bromo-3-methylsulfinyl-1-benzofuran-2-yl)acetate (Choi *et al.*, 2008*b*).  Here we report the crystal structure of the title compound, methyl 2-(5-fluoro-3-methylsulfinyl-1-benzofuran-2-yl)acetate (Fig. 1).

The benzofuran unit is essentially planar, with a mean deviation of 0.006 (1) ° from the least-squares plane defined by the nine constituent atoms (Fig. 1).  In the title compound, all of the bond angles and bond distances are in the normal range of related molecules (Choi *et al.*, 2008*a, b*).  The oxygen atom and the methyl group of the methylsulfinyl substituent lie on opposite sides of the plane of the benzofuran fragment [O4–S–C1–C8 and C12–S–C1–C8 torsional angles = 126.70 (13)° and -123.55 (13) °, respectively].  Crystal packing  is stabilized by weak intermolecular C–H···O hydrogen bond interactions involving the S═O unit with the benzofurn ring (C3–H3···O4^i^) and a methylene H atom (C9–H9A···O4^ii^), between the furan ring and methylene H atom (C9–H9B···O1^iii^) as well as between a methyl H atom of the methylsulfinyl substituent and the C═O unit (C12–H12A···O3^iv^), respectively (Fig. 2, Table 1).  Crystal packing also exhibits aromatic π–π stacking interactions between the furan/benzene and the benzene/benzene rings of neighbouring molecules [Cg1···Cg2^vii^ = 3.8258 (9)Å and Cg2···Cg2^vi^ = 3.8794 (9)Å, where Cg1 and Cg2 are centroids of the furan (C1/C2/C7/O1/C8) and benzene (C2-C7) rings, respectively, Fig. 3].  The molecular packing is further stabilized by a weak, intermolecular C–H···π ring interaction between a methyl H atom of the methoxy group and a benzene ring of a neighbouring molecule (C11–H11A···Cg2^v^; = 3.927 (3)Å, Table 1).

### Experimental

The 77% 3-chloroperoxybenzoic acid (247 mg, 1.1 mmol) was added in small portions to a stirred solution of methyl 2-(5-fluoro-3-methylsulfanyl-1-benzofuran-2-yl)acetate (254 mg, 1.0 mmol) in dichloromethane (30 ml) at 273 K. After being stirred for 4 h at room temperature, the mixture was washed with saturated sodium bicarbonate solution and the organic layer was separated, dried over magnesium sulfate, filtered and concentrated in vacuum. The residue was purified by column chromatography (hexane-ethyl acetate,1:2 v/v) to afford the title compound as a colorless solid [yield 79%, m.p. 370-371 K; R_f_ = 0.4 (hexane-ethyl acetate, 1;2 v/v )]. Single crystals suitable for X-ray diffraction were prepared by slow evaporation of a solution of the title compound in benzene at room temperature.

### Refinement

All H atoms were geometrically positioned and refined using a riding model, with C–H = 0.93 Å for the aryl, 0.97 Å for the methylene, and 0.96 Å for the methyl H atoms. *U*_iso_(H) = 1.2 *U*_eq_(C) for the aryl and methylene H atoms, and 1.5 *U*_eq_(C) for methyl H atoms.

### Crystal data

**Table:**

C_12_H_11_FO_4_S	*Z* = 2
*M**_r_* = 270.27	*F*(000) = 280
Triclinic, *P*1	*D*_x_ = 1.442 Mg m^−^^3^
Hall symbol: -P 1	Mo *K*α radiation, λ = 0.71073 Å
*a* = 7.7799 (5) Å	Cell parameters from 3896 reflections
*b* = 8.5609 (6) Å	θ = 2.7–27.5°
*c* = 10.5592 (7) Å	µ = 0.28 mm^−^^1^
α = 73.834 (1)°	*T* = 273 K
β = 80.178 (1)°	Block, colorless
γ = 67.486 (1)°	0.60 × 0.40 × 0.40 mm
*V* = 622.36 (7) Å^3^	

### Data collection

**Table:**

Bruker SMART CCD diffractometer	2389 reflections with *I* > 2σ(*I*)
Radiation source: fine-focus sealed tube	*R*_int_ = 0.014
graphite	θ_max_ = 27.0°, θ_min_ = 2.0°
Detector resolution: 10.0 pixels mm^-1^	*h* = −9→9
φ and ω scans	*k* = −10→10
5368 measured reflections	*l* = −13→13
2667 independent reflections	

### Refinement

**Table:**

Refinement on *F*^2^	Primary atom site location: structure-invariant direct methods
Least-squares matrix: full	Secondary atom site location: difference Fourier map
*R*[*F*^2^ > 2σ(*F*^2^)] = 0.032	Hydrogen site location: difference Fourier map
*wR*(*F*^2^) = 0.089	H-atom parameters constrained
*S* = 1.08	*w* = 1/[σ^2^(*F*_o_^2^) + (0.0432*P*)^2^ + 0.227*P*] where *P* = (*F*_o_^2^ + 2*F*_c_^2^)/3
2667 reflections	(Δ/σ)_max_ = 0.001
165 parameters	Δρ_max_ = 0.28 e Å^−^^3^
0 restraints	Δρ_min_ = −0.31 e Å^−^^3^

### Special details

**Table:**

Geometry. All esds (except the esd in the dihedral angle between two l.s. planes) are estimated using the full covariance matrix. The cell esds are taken into account individually in the estimation of esds in distances, angles and torsion angles; correlations between esds in cell parameters are only used when they are defined by crystal symmetry. An approximate (isotropic) treatment of cell esds is used for estimating esds involving l.s. planes.
Refinement. Refinement of *F*^2^ against ALL reflections. The weighted *R*-factor *wR* and goodness of fit *S* are based on *F*^2^, conventional *R*-factors *R* are based on *F*, with *F* set to zero for negative *F*^2^. The threshold expression of *F*^2^ > σ(*F*^2^) is used only for calculating *R*-factors(gt) *etc*. and is not relevant to the choice of reflections for refinement. *R*-factors based on *F*^2^ are statistically about twice as large as those based on *F*, and *R*- factors based on ALL data will be even larger.

### Fractional atomic coordinates and isotropic or equivalent isotropic displacement parameters (Å^2^)

**Table:**

	*x*	*y*	*z*	*U*_iso_*/*U*_eq_	
S	0.73212 (5)	0.63256 (5)	0.04928 (3)	0.03039 (12)	
F	0.30035 (14)	0.21264 (13)	0.37311 (11)	0.0513 (3)	
O1	0.84250 (14)	0.43881 (13)	0.42317 (9)	0.0288 (2)	
O2	1.04499 (16)	0.88742 (15)	0.21928 (13)	0.0436 (3)	
O3	0.75373 (16)	0.90527 (15)	0.20563 (13)	0.0449 (3)	
O4	0.75098 (17)	0.49556 (16)	−0.02040 (11)	0.0415 (3)	
C1	0.73869 (19)	0.53270 (18)	0.21918 (13)	0.0263 (3)	
C2	0.63718 (19)	0.42498 (17)	0.29942 (13)	0.0261 (3)	
C3	0.4978 (2)	0.37061 (19)	0.27953 (15)	0.0309 (3)	
H3	0.4480	0.4036	0.1981	0.037*	
C4	0.4390 (2)	0.26551 (19)	0.38775 (17)	0.0352 (3)	
C5	0.5098 (2)	0.2096 (2)	0.51151 (16)	0.0375 (4)	
H5	0.4641	0.1377	0.5804	0.045*	
C6	0.6489 (2)	0.26217 (19)	0.53113 (15)	0.0342 (3)	
H6	0.7000	0.2268	0.6124	0.041*	
C7	0.70799 (19)	0.36995 (18)	0.42381 (14)	0.0275 (3)	
C8	0.85804 (19)	0.53640 (18)	0.29708 (13)	0.0264 (3)	
C9	0.9967 (2)	0.62392 (19)	0.27115 (14)	0.0290 (3)	
H9A	1.0886	0.5816	0.2024	0.035*	
H9B	1.0611	0.5919	0.3506	0.035*	
C10	0.9129 (2)	0.82024 (19)	0.22925 (14)	0.0296 (3)	
C11	0.9865 (3)	1.0754 (2)	0.1780 (3)	0.0657 (6)	
H11A	0.9333	1.1147	0.0941	0.099*	
H11B	1.0924	1.1099	0.1701	0.099*	
H11C	0.8950	1.1261	0.2425	0.099*	
C12	0.4904 (2)	0.7705 (2)	0.04906 (17)	0.0386 (4)	
H12A	0.4625	0.8393	−0.0388	0.058*	
H12B	0.4656	0.8458	0.1077	0.058*	
H12C	0.4139	0.7000	0.0782	0.058*	

### Atomic displacement parameters (Å^2^)

**Table:**

	*U*^11^	*U*^22^	*U*^33^	*U*^12^	*U*^13^	*U*^23^
S	0.0313 (2)	0.0392 (2)	0.02016 (18)	−0.01352 (15)	−0.00616 (13)	−0.00224 (14)
F	0.0428 (6)	0.0440 (6)	0.0702 (7)	−0.0247 (5)	−0.0127 (5)	0.0003 (5)
O1	0.0340 (5)	0.0313 (5)	0.0211 (5)	−0.0111 (4)	−0.0076 (4)	−0.0034 (4)
O2	0.0324 (6)	0.0323 (6)	0.0661 (8)	−0.0123 (5)	−0.0036 (5)	−0.0104 (5)
O3	0.0323 (6)	0.0356 (6)	0.0588 (8)	−0.0086 (5)	−0.0120 (5)	0.0019 (5)
O4	0.0454 (7)	0.0528 (7)	0.0277 (6)	−0.0129 (5)	−0.0069 (5)	−0.0154 (5)
C1	0.0278 (7)	0.0284 (7)	0.0209 (6)	−0.0074 (5)	−0.0053 (5)	−0.0046 (5)
C2	0.0270 (7)	0.0249 (6)	0.0237 (6)	−0.0050 (5)	−0.0036 (5)	−0.0065 (5)
C3	0.0294 (7)	0.0285 (7)	0.0334 (7)	−0.0070 (6)	−0.0071 (6)	−0.0069 (6)
C4	0.0295 (7)	0.0278 (7)	0.0477 (9)	−0.0100 (6)	−0.0051 (6)	−0.0065 (6)
C5	0.0389 (8)	0.0275 (7)	0.0382 (8)	−0.0103 (6)	0.0018 (6)	0.0000 (6)
C6	0.0403 (8)	0.0299 (7)	0.0261 (7)	−0.0082 (6)	−0.0043 (6)	−0.0017 (6)
C7	0.0283 (7)	0.0267 (7)	0.0261 (7)	−0.0065 (5)	−0.0045 (5)	−0.0070 (5)
C8	0.0276 (7)	0.0264 (7)	0.0217 (6)	−0.0055 (5)	−0.0048 (5)	−0.0041 (5)
C9	0.0265 (7)	0.0315 (7)	0.0285 (7)	−0.0078 (6)	−0.0080 (5)	−0.0061 (5)
C10	0.0302 (7)	0.0338 (7)	0.0238 (7)	−0.0112 (6)	−0.0019 (5)	−0.0055 (5)
C11	0.0528 (12)	0.0333 (9)	0.1083 (19)	−0.0182 (9)	−0.0025 (12)	−0.0103 (10)
C12	0.0349 (8)	0.0362 (8)	0.0382 (8)	−0.0074 (6)	−0.0134 (6)	0.0001 (6)

### Geometric parameters (Å, °)

**Table:**

S—O4	1.500 (2)	C4—C5	1.391 (2)
S—C1	1.760 (1)	C5—C6	1.385 (2)
S—C12	1.797 (2)	C5—H5	0.9300
F—C4	1.365 (2)	C6—C7	1.383 (2)
O1—C8	1.374 (2)	C6—H6	0.9300
O1—C7	1.382 (2)	C8—C9	1.484 (2)
O2—C10	1.335 (2)	C9—C10	1.514 (2)
O2—C11	1.450 (2)	C9—H9A	0.9700
O3—C10	1.200 (2)	C9—H9B	0.9700
C1—C8	1.355 (2)	C11—H11A	0.9600
C1—C2	1.444 (2)	C11—H11B	0.9600
C2—C7	1.398 (2)	C11—H11C	0.9600
C2—C3	1.398 (2)	C12—H12A	0.9600
C3—C4	1.374 (2)	C12—H12B	0.9600
C3—H3	0.9300	C12—H12C	0.9600
			
O4—S—C1	106.58 (7)	C6—C7—C2	123.8 (1)
O4—S—C12	106.22 (8)	C1—C8—O1	111.2 (1)
C1—S—C12	98.29 (7)	C1—C8—C9	132.4 (1)
C8—O1—C7	106.3 (1)	O1—C8—C9	116.5 (1)
C10—O2—C11	116.0 (1)	C8—C9—C10	114.0 (1)
C8—C1—C2	107.3 (1)	C8—C9—H9A	108.8
C8—C1—S	123.2 (1)	C10—C9—H9A	108.8
C2—C1—S	129.4 (1)	C8—C9—H9B	108.8
C7—C2—C3	119.5 (1)	C10—C9—H9B	108.8
C7—C2—C1	104.8 (1)	H9A—C9—H9B	107.7
C3—C2—C1	135.8 (1)	O3—C10—O2	124.1 (1)
C4—C3—C2	115.9 (1)	O3—C10—C9	126.4 (1)
C4—C3—H3	122.1	O2—C10—C9	109.5 (1)
C2—C3—H3	122.1	O2—C11—H11A	109.5
F—C4—C3	117.7 (1)	O2—C11—H11B	109.5
F—C4—C5	117.5 (1)	H11A—C11—H11B	109.5
C3—C4—C5	124.8 (2)	O2—C11—H11C	109.5
C6—C5—C4	119.4 (1)	H11A—C11—H11C	109.5
C6—C5—H5	120.3	H11B—C11—H11C	109.5
C4—C5—H5	120.3	S—C12—H12A	109.5
C7—C6—C5	116.6 (1)	S—C12—H12B	109.5
C7—C6—H6	121.7	H12A—C12—H12B	109.5
C5—C6—H6	121.7	S—C12—H12C	109.5
O1—C7—C6	125.7 (1)	H12A—C12—H12C	109.5
O1—C7—C2	110.5 (1)	H12B—C12—H12C	109.5
			
O4—S—C1—C8	126.70 (13)	C5—C6—C7—C2	−0.8 (2)
C12—S—C1—C8	−123.55 (13)	C3—C2—C7—O1	−179.47 (12)
O4—S—C1—C2	−48.22 (14)	C1—C2—C7—O1	0.62 (15)
C12—S—C1—C2	61.53 (14)	C3—C2—C7—C6	0.3 (2)
C8—C1—C2—C7	−0.35 (15)	C1—C2—C7—C6	−179.62 (13)
S—C1—C2—C7	175.19 (11)	C2—C1—C8—O1	−0.04 (16)
C8—C1—C2—C3	179.77 (15)	S—C1—C8—O1	−175.92 (9)
S—C1—C2—C3	−4.7 (2)	C2—C1—C8—C9	−179.96 (14)
C7—C2—C3—C4	0.6 (2)	S—C1—C8—C9	4.2 (2)
C1—C2—C3—C4	−179.54 (15)	C7—O1—C8—C1	0.42 (15)
C2—C3—C4—F	178.65 (12)	C7—O1—C8—C9	−179.65 (12)
C2—C3—C4—C5	−1.0 (2)	C1—C8—C9—C10	61.1 (2)
F—C4—C5—C6	−179.15 (14)	O1—C8—C9—C10	−118.82 (13)
C3—C4—C5—C6	0.5 (2)	C11—O2—C10—O3	0.5 (2)
C4—C5—C6—C7	0.4 (2)	C11—O2—C10—C9	179.20 (16)
C8—O1—C7—C6	179.59 (14)	C8—C9—C10—O3	−6.6 (2)
C8—O1—C7—C2	−0.65 (14)	C8—C9—C10—O2	174.75 (12)
C5—C6—C7—O1	178.93 (13)		

### Hydrogen-bond geometry (Å, °)

**Table:**

*D*—H···*A*	*D*—H	H···*A*	*D*···*A*	*D*—H···*A*
C3—H3···O4^i^	0.93	2.39	3.303 (2)	166
C9—H9A···O4^ii^	0.97	2.22	3.179 (2)	168
C9—H9B···O1^iii^	0.97	2.54	3.489 (2)	166
C12—H12A···O3^iv^	0.96	2.60	3.478 (2)	152
C11—H11A···Cg2^v^	0.96	2.97	3.93	173

Symmetry codes: (i) −*x*+1, −*y*+1, −*z*; (ii) −*x*+2, −*y*+1, −*z*; (iii) −*x*+2, −*y*+1, −*z*+1; (iv) −*x*+1, −*y*+2, −*z*; (v) *x*, *y*+1, *z*.
